# The Application of Internet-Based Sources for Public Health Surveillance (Infoveillance): Systematic Review

**DOI:** 10.2196/13680

**Published:** 2020-03-13

**Authors:** Joana M Barros, Jim Duggan, Dietrich Rebholz-Schuhmann

**Affiliations:** 1 Insight Centre for Data Analytics National University of Ireland Galway Galway Ireland; 2 School of Computer Science National University of Ireland Galway Galway Ireland; 3 ZB MED - Information Centre for Life Sciences University Cologne Cologne Germany

**Keywords:** medical informatics, public health informatics, public health, infectious diseases, chronic diseases, infodemiology, infoveillance

## Abstract

**Background:**

Public health surveillance is based on the continuous and systematic collection, analysis, and interpretation of data. This informs the development of early warning systems to monitor epidemics and documents the impact of intervention measures. The introduction of digital data sources, and specifically sources available on the internet, has impacted the field of public health surveillance. New opportunities enabled by the underlying availability and scale of internet-based sources (IBSs) have paved the way for novel approaches for disease surveillance, exploration of health communities, and the study of epidemic dynamics. This field and approach is also known as infodemiology or infoveillance.

**Objective:**

This review aimed to assess research findings regarding the application of IBSs for public health surveillance (infodemiology or infoveillance). To achieve this, we have presented a comprehensive systematic literature review with a focus on these sources and their limitations, the diseases targeted, and commonly applied methods.

**Methods:**

A systematic literature review was conducted targeting publications between 2012 and 2018 that leveraged IBSs for public health surveillance, outbreak forecasting, disease characterization, diagnosis prediction, content analysis, and health-topic identification. The search results were filtered according to previously defined inclusion and exclusion criteria.

**Results:**

Spanning a total of 162 publications, we determined infectious diseases to be the preferred case study (108/162, 66.7%). Of the eight categories of IBSs (search queries, social media, news, discussion forums, websites, web encyclopedia, and online obituaries), search queries and social media were applied in 95.1% (154/162) of the reviewed publications. We also identified limitations in representativeness and biased user age groups, as well as high susceptibility to media events by search queries, social media, and web encyclopedias.

**Conclusions:**

IBSs are a valuable proxy to study illnesses affecting the general population; however, it is important to characterize which diseases are best suited for the available sources; the literature shows that the level of engagement among online platforms can be a potential indicator. There is a necessity to understand the population’s online behavior; in addition, the exploration of health information dissemination and its content is significantly unexplored. With this information, we can understand how the population communicates about illnesses online and, in the process, benefit public health.

## Introduction

### Background

Public health is “the art and science of preventing disease, prolonging life and promoting health through the organized efforts of society” [1]. As a research and political field, it is focused on improving the quality of life of the population by identifying, suggesting, and applying prevention measures (eg, through the promotion of healthy behaviors) and health-related treatments [2]. Monitoring health is one important contribution to public health measures and involves the systematic collection, analysis, and interpretation of large amounts of health-related data. The key aim of public health surveillance is to design and guide interventions; in particular, (1) it serves as an early warning system for health emergencies (*epidemics*, ie, acute events), (2) it documents public health interventions and tracks their progress (ie, *monitoring health*), and (3) it monitors and clarifies the epidemiology of health problems, enabling the prioritization of information necessary for the formulation of health policy (ie, targeting chronic events) [3].

In the past, surveillance has been based on reports from health care workers constituting an active surveillance system when consistent and standardized reporting is in place [3,4]. However, this architecture is costly to maintain and involves significant delays between the moment of data capture to the time point of the first diagnosis, thus hampering any rapid or even immediate detection of outbreaks [5]. Instead of attempting to gather surveillance data from a network of health facilities and laboratories, health entities can employ a passive surveillance system in which hospitals, clinics, or other similar sources submit their respective health reports. This system provides an inexpensive way to monitor the community’s health; however, data quality is an issue owing to nonuniform reporting standards, and timeliness remains difficult to achieve [4]. To further complement these systems, syndromic surveillance was created to deal with the timeliness issue by using clinical (eg, emergency department admissions) and nonclinical sources (eg, over-the-counter drug sales), which are available before a diagnosis is confirmed [4]. This type of surveillance is based on the assumption that an outbreak would manifest itself as an anomaly in normal behavior [5]. In line with syndromic surveillance and with the growth of the internet, new opportunities for the detection of health-related information have arisen, with the potential to capture the patient’s input directly from the source. This leads to the ambitious endeavor of being able to monitor the health of a significant portion of the population at any point in time and at any geographical location, with the ultimate goal of monitoring public health.

The abovementioned technological advancements have enabled unofficial informal sources to currently provide more than 60% of epidemic reports [6]. Data analytics based on these data sources can provide near real-time outbreak information in various formats (independently from official governmental output) and have been successfully tested for health-related purposes. Furthermore, these sources offer the unique advantage of providing firsthand evidence for occurrences of health-related events (eg, through social media channels) and real-time informal reports (eg, news), which can be immediately investigated. Any analysis can be focused only on continuous monitoring, or by contrast to the identification of specific events (ie, single disease focus). In the latter case, it can be targeted to identify isolated hints (eg, mentions of flu) or to determine significant changes in public reporting; it can be further extended to consider the location of the population at risk or to monitor the distribution or extension of an epidemic (eg, influenced by the travelling population). The potential of data analytics applied to public data for health-related developments is ever more far-reaching in our increasingly digitally equipped society; thus, these approaches have an important role in the improvement of timeliness and sensitivity (ie, rapidly and correctly identifying health mentions) in public health surveillance [7].

### Internet-Based Sources for Public Health Surveillance

IBSs are characterized by providing unstructured information from multiple origins and have proven to detect the first evidence of an outbreak, which is particularly beneficial for locations with a limited capacity for public health surveillance. The use of these sources for public health is also known as infodemiology or infoveillance. With the evidence provided by these sources, health agents are capable of mobilizing rapid response, reducing morbidity and mortality [8,9]. Some examples of IBSs include search queries, web encyclopedias, microblogs, and other social media.

Infectious diseases became the initial case study for the application of IBSs for disease surveillance. These continue to be a major cause of death in low-income countries [10], with research initially focusing on dengue, and are responsible for recurrent threats in the rest of the world (eg, *swine flu* and *bird flu*). Furthermore, these diseases are continuously monitored by official sources through laboratory tests or sentinel systems over many years and such information now forms the ground-truth data used to validate the findings from IBSs [11].

ProMED-mail is one of the first applications of such sources. This system is currently used for communication, via email and reports, among the infectious disease community [12]. Other systems include aggregators such as Global Public Health Intelligence Network, BioCaster, and HealthMap. These initially targeted a variety of sources including emails, Really Simple Syndication feeds, and PDF documents to extract information referring to an increased number of clusters of infected people at a specified time, period, or location, which could indicate a threat. The aggregator systems still in operation also include additional sources such as social media [7,13]. Moving to other sources, influenza-like illnesses (ILIs) served as a prototypical case study owing to being seasonal, worldwide, and well-reported diseases and initiated the monitoring of web-based queries. In particular, one of the first studies utilized Google search volumes to estimate the percentage of ILI-related physician visits [14]. This source was further adapted to the surveillance of other diseases such as dengue [15], gastroenteritis, and chickenpox [16]. This initial success led Google to develop targeted tools for the monitoring of influenza (Google Flu Trends) and dengue (Google Dengue Trends) in 2008 and 2011, respectively, which were later discontinued. Research continued and aimed to identify the most appropriate search terms to utilize as well as other search services (eg, Yahoo [17]) and other languages (eg, Vårdguiden [18]). Following search queries, microblogs, in particular, Twitter, showed to be another source of health information characterized by providing more descriptive information and potentially containing semistructured metadata (eg, location and gender) [19,20]. By filtering messages containing disease-related keywords, the frequency of disease mentions can be tracked and outbreaks can be identified as unusual spikes in the message frequency [21]. Similar to search queries, subsequent research focused on the identification of adequate keywords, as well as the identification of personal health messages, ie, containing a keyword relevant to the disease and describing a first-person infection case, among others [22,23]. With the use of more descriptive albeit semantically ambiguous data, the focus shifted to detecting true signals, ie, first-person occurrences of diseases. The application of IBSs continued to grow [24] in tandem with the addition of new sources such as Facebook [25], Instagram [26], and discussion forums [27]. Noncommunicable diseases (NCDs) are the cause of 71% of deaths globally, ranging from 37% in low-income countries to 88% in high-income countries, hence, internet-based surveillance focus has begun to also include NCDs [10]. In this case, emphasis was given to the online behavior of affected individuals, as well as to the content of the information present in the sources [28], with the goal to establish or improve health practices and support the dissemination of health information to address the needs of the population [29,30].

Owing to the unstructured nature and to the large volumes of data provided by these IBSs, tailoring of solutions, applications, and even tools for retrieving and filtering the content becomes vital for success. Subsequent automatic use of these methods then becomes the key step to monitor the internet sources continuously, and eventually to identify potential public health risks or, even better, risks to individual patients [31]. However, disease surveillance based on online sources must be used with caution. Automatic identification of disease events from web-based data streams has to cope with inherent biases, ie, false-positive events, introduced through geographic or cultural variability in language and reporting when compared with reliable traditional surveillance methods [32]. Furthermore, traditional epidemiological parameters (eg, attack rate) are often not available as a gold standard and thus limit the proper assessment of the applied methods [31].

### Objectives

Our objective was to provide a discriminative assessment of the applications of IBSs for disease surveillance and their use as ground truth for future research. To achieve this, we have presented a comprehensive systematic literature review with a focus on IBSs and their limitations, the diseases targeted, and methods commonly applied for disease surveillance. Our research questions (RQs) were as follows:

1. What internet-based sources are utilized for infoveillance and infodemiology?
2. What is the aim of the research conducted using these sources?
3. How are internet-based sources applied to generate knowledge?
4. Which sources have shown a preference for studying communicable and noncommunicable diseases?
5. What are the common limitations of internet-based sources?

## Methods

### Search Strategy

**Figure 1:**
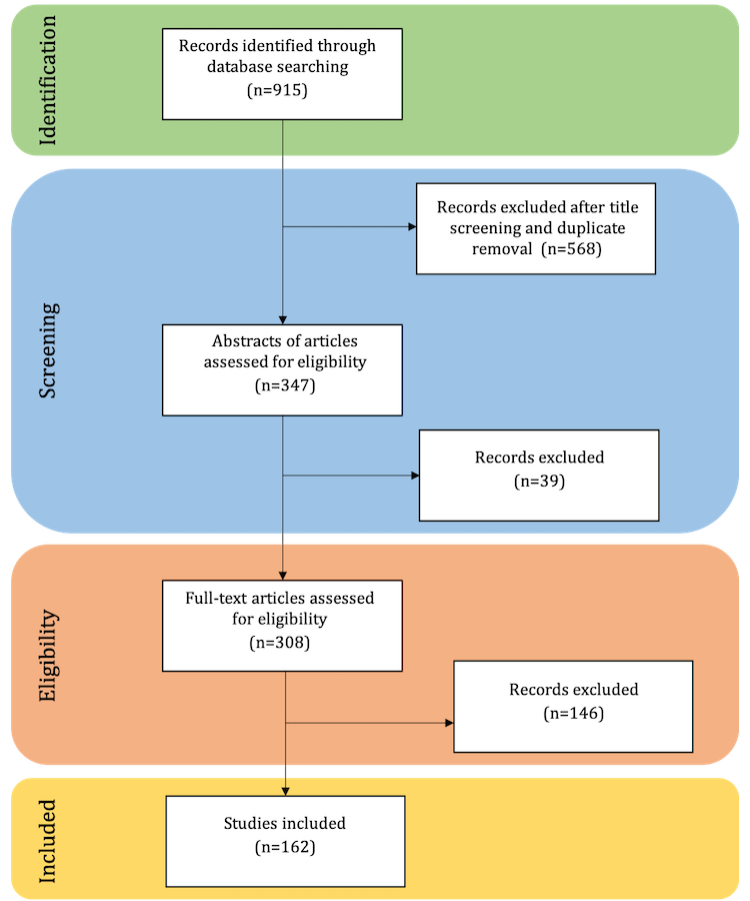
Flowchart applied for the literature search.

This review was conducted through several stages based on the Preferred Reporting Items for Systematic Reviews and Meta-Analyses process (Figure 1). To be the most inclusive, eight mutually exclusive research libraries that contain a variety of journals in the fields of Informatics and Biomedical Sciences were selected. The libraries were Europe PubMed Central, Institute of Electrical and Electronics Engineers Xplore Digital Library, Association for Computing Machinery Digital Library, SpringerLink, EBSCO Host, PubMed, Scopus, and Web of Science. Keyword generation was focused on IBSs of public health data, infoveillance, infodemiology, and disease outbreak and surveillance. We considered all conference and journal articles identified in this process.

### Article Selection

The keywords were generated taking into consideration a preliminary assessment of the literature through a manual screening of relevant studies to ensure the list was complete. The complete list of these keywords can be found in Multimedia Appendix 1. The literature search was initially performed from October 10 to October 31, 2017, on the abovementioned repositories, focusing on the publication period of 2012 to 2017. This literature search was later augmented to include additional search terms and to extend the publication period until 2018. In total, the literature search had a duration of 2 months, excluding the screening and eligibility steps. Our review focused on the literature published after 2012 to cover a wider variety of sources, given the time lag between their popularity peaks; furthermore, by analyzing the literature published a few years after the first studies (eg, 2009 for search queries [14] and 2010 for social media [19]), we focused on research with finer-grained and adapted methodologies (eg, improved keyword selection and relevancy filters). To select relevant articles, a multiphase process was implemented. First, the article title was screened for relevance and duplicates were removed; subsequently the abstract was screened, and only articles that passed both steps were considered for the eligibility phase. The inclusion and exclusion criteria for the articles were decided according to a modified PICOTS. The criteria are specified in Multimedia Appendix 1. The first author performed the screening process and retrieved the data. When doubts were raised regarding the inclusion of certain publications, the remaining authors were consulted.

### Quality Assessment

To address the quality of the studies, we implemented a set of criteria to evaluate the publications retrieved. This assessment was based on a set of questions with regard to the purpose of research, contextualization, methodology, study design, the results obtained, and findings. The quality criteria are based on the work by Kofod-Petersen [33] and are present in Multimedia Appendix 1.

### Data Extraction

We also developed an extraction form to gather information about the studies allowing us to understand how the issues related to the proposed RQs have been addressed. This step was performed using the NVivo version 11 qualitative software database (QSR International Pty Ltd), nested *cases* were used to annotate each item of the extraction form. The extraction guidelines are available in Multimedia Appendix 1. For each checklist item in the guidelines, we created a classification that has been detailed in the following sections. Each paper was classified as *journal* or *conference*, in accordance with the inclusion criteria. Regarding the targeted diseases, we divided this into *chronic*, *infectious*, *medical conditions*, and *health topics*. The first three categories have been further specified. For the Goal/Objective item, a paper was classified as *outbreak forecasting* if it explicitly stated that the research was aimed at forecasting; else, it was assigned to *surveillance* (ie, when the purpose is only to identify the degree of correlation with ground-truth data and there is no mention of forecasting); *disease characterisation* was assigned when the aim was to determine identifiable characteristics related to a disease, eg, patient search behavior and commonly mentioned treatments, or when the aim was to classify a text as related to a disease; *content analysis* was assigned to the study of the sources’ content (eg, news presence and expressed sentiment) referring to a disease or medical condition; *personal health mention classification* focused on separating general mentions of a disease or medical conditions (eg, news) from first-person mentions; and *diagnosis prediction* was assigned when the purpose was to attribute a disease or medical condition to a text and its creator by proxy. The *Internet-based data source* can be classified into search queries, social media (including microblogs), websites, news, discussion forums, web encyclopedia, and media monitoring systems. We also considered the use of data sources external to the IBSs, which can be classified as demographic, socioeconomic, and climate statistics, as well as data from governmental and laboratory sources, among others. To address the study design/methodology, we devised the following criteria: *topic analysis* corresponds to when topic modelling and similar approaches are used; *regression models* encompass all regression and autoregression models (eg, linear regression and autoregressive moving average); *statistical models* was assigned to more complex models (eg, Hidden Markov Chain); *correlation analysis* was used when correlation scores are calculated (eg, Pearson); *rule-based techniques* and *ranking techniques* are self-explanatory; *manual analysis* was assigned when no specific techniques are used other than a manual assessment; *epidemiology theory* refers to the use of techniques and measures commonly used in epidemiology (eg, Susceptible, Exposed, Infectious, and Recovered models); *linguistic analysis* was assigned when sentiment analysis and lexicons, among others, were used; and finally, we split *machine learning* and *deep learning*. Finally, we did not add a classification to the findings and limitations; we chose to keep this as an open field and manually analyzed the outcomes.

## Results

### Overview

**Figure 2:**
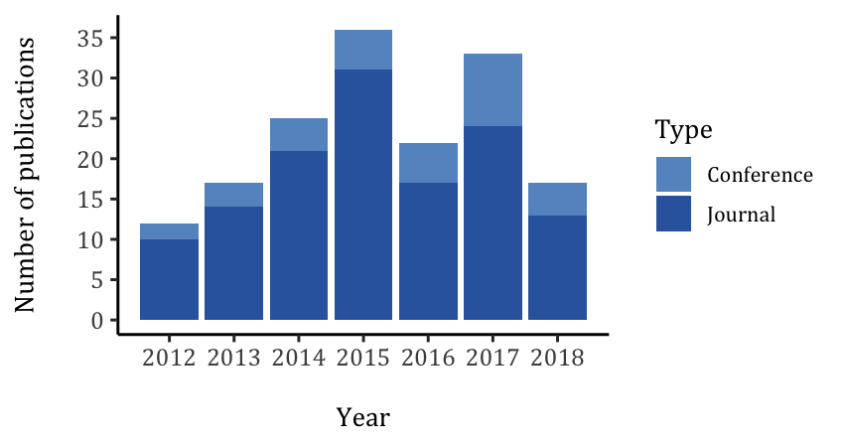
Distribution of the selected literature per year and type.

The results from the search strategy are shown in Figure 1; in total, 162 papers were considered for this systematic literature review. The summary of the review results according to the data extraction guidelines is presented in Multimedia Appendix 1 [34-188]. The year with the highest number of publications is 2015 (n=36), followed by 2017 (n=33), 2014 (n=25), 2016 (n=22), 2013 (n=17), 2018 (n=17), and 2012 (n=12; Figure 2). Journal articles accounted for 130 of all publications and the remaining 32, for conferences. The remaining results were split into subsections correspondent to the extraction guidelines followed. The complete summary of the literature analysis is provided in Multimedia Appendix 1.

### Goal

The analyzed papers mostly focused on surveillance (n=90), content analysis (n=46), and outbreak forecasting (n=45); other goals included personal health mention classification (n=10), disease characterization (n=5), and diagnosis prediction (n=4), with 36 papers having multiple targets.

### Diseases, Medical Conditions, and Health Topics

**Figure 3:**
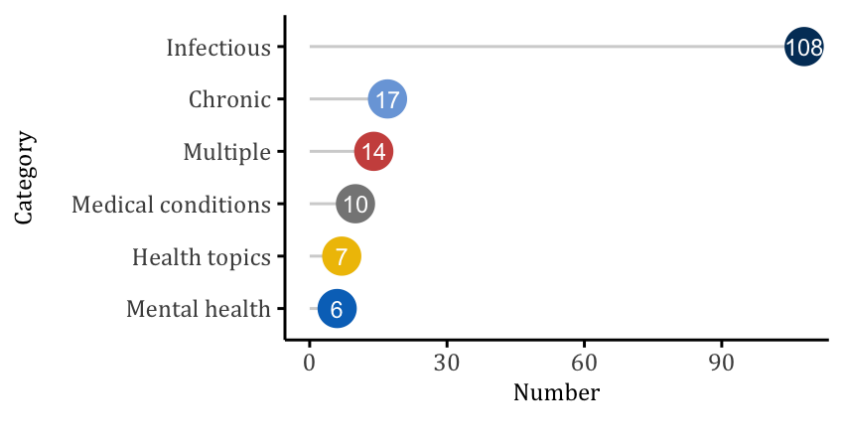
Distribution of the case studies in the literature.

Infectious diseases are markedly the most researched cases, with a total of 108 papers assigned. Chronic diseases are the focus of 17 publications, followed by medical conditions with 10 publications, health topics with 7 mentions, and mental health with 6 assigned articles (Figure 3). A set of 14 publications target multiple diseases from all the previously mentioned categories. Among the infectious diseases, ILIs, dengue, and infectious intestinal diseases are the top choices with 57, 7, and 7 assigned publications, respectively. In terms of chronic illnesses, cancer is the most researched disease (n=3). Excluding the publications focusing on multiple cases, 78% of the diseases appear in less than two articles.

### Internet-Based and External Data Sources

The 162 analyzed papers can be classified into eight distinct categories: search queries, social media, news, discussion forums, websites, media monitoring systems, web encyclopedia, and online obituaries. Social media (n=80) and search queries (n=79) are the most utilized IBSs, followed by web encyclopedias (n=13) that, in the selected papers, corresponded solely to Wikipedia. The remaining are utilized in the following decreasing order, forums (n=9), news (n=8), media monitoring systems (n=2), online obituaries (n=2), and websites not related to newspapers (n=1). A total of 29 papers utilize combinations of these sources, with the majority (n=11) combining search queries with social media. For social media, Twitter is mostly used (n=71) with the remaining sources marginally appearing. For search queries, the same behavior is seen with Google Trends; it is present in 42 publications, and when aggregating with Google Flu Trends and Google Dengue Trends, this value rises to 61.

Regarding sources external to IBSs, governmental, or laboratory surveillance statistics are the most utilized and used as ground-truth data (n=107), the second most used external source is hospital and emergency department visits (n=16), which are also used as ground-truth data. Climate or temperature statistics are applied in 8 papers, and socioeconomic statistics in 5 publications followed by health records in 4 publications, demographic or population statistics in 3 papers, and pharmaceutical sales in 2 publications. Scientific search engines, Flu Near You [189], and telephone triage are used individually in only 1 publication. In total, 45 publications do not use any external data source, and 26 publications share multiple external sources.

### Study Design

Regarding the methodologies used, correlation analysis (n=59) was predominant and closely followed by regression models (n=46). Machine learning was used in 27 of the analyzed articles, statistical models are preferred in 20 publications, manual analysis was used in 18 of the articles, topic analysis is used in 12 publications, and deep learning and linguistic analysis were used in 10 articles each. Regarding the remaining methodology, rule-based techniques (n=7), epidemiology theory (n=6), surveys (n=3), and ranking techniques (n=1) were used in less than 10 papers.

### Findings and Limitations

Qualitatively, the studies reported positive results (n=125), mentioning high or improved correlations with ground-truth data, as well as the outbreak predictive power, and high accuracy when the goal was surveillance, outbreak forecasting, personal health mention classification, disease characterization, and diagnosis prediction. The studies by Olson et al [55], Alicino et al [85], and Yom-tov [109] report negative results caused by questionable reliability with search query data and media influence affecting social networks and web encyclopedias. For the publications solely focused on content analysis (n=30), the findings were reported without a negative or positive association. A total of four publications [54,91,115,130] mention positive and negative results related to a large variation in the correlation score with social network data, surveillance inaccuracies for different age groups and the lack of specificity for search query data, and media influence when applying both social network and search query data.

In terms of limitations, these can be divided into gold standard (n=22); representativeness (n=76); general bias, eg, change in search behavior, symptom variability (n=9), and media effect (n=17); dataset size (n=7); methodology, eg, computational cost, keywords, and spelling errors (n=63); language (n=11); geographical restriction (n=33); and timeframe restriction (n=20).

## Discussion

In this study, we aimed to provide a discriminative assessment on the application of IBSs in public health. To achieve this, we focused on the literature published in the last 6 years and applied systematic selection criteria to determine the appropriate studies to include. As a result, we proposed a taxonomy and identified the gaps to be addressed in future research, represented by the identified limitations of IBSs. Hence, this section addresses each RQ stated in the objectives.

### Research Question 1: What Internet-Based Sources Are Utilized for Infoveillance and Infodemiology?

As reported in the Results, search queries, social media, discussion forums, news, web encyclopedia, online obituaries, media monitoring systems, and websites constitute the general categories of the IBSs present in the analyzed literature.

For search queries and social media, there is a large variation in the sources, which is mostly caused by geographical differences. The sources include platforms that are only available to certain countries. In the case of search queries, this potentially brings benefits in representativeness as it is possible to estimate the country-wide disease surveillance data from online search behavior. We argue that using a worldwide search engine, cultural differences that shape online search behavior could be diluted further complicating the surveillance task. Google Trends and its variations are the most common and widely represented; Bing also has an extensive geographical representation but a lower market share [190]. Also included are Baidu, Naver, Yandex, Vårdguiden and Websök, and Sapo, which cater to different countries, namely China, South Korea, Russia, Sweden, and Portugal, respectively. Nonetheless, the use of country-specific search engines can be limited by their market share, as is the case for Sapo [191], and their fine-grained geographical representation, eg, Vårdguiden is mostly used by people in Stockholm [68]. These limitations are further discussed in the following subsections.

Regarding social media, the sources differ in content richness. For example, while Twitter is a microblogging service, Weibo incorporates functionalities that can also be found on Facebook [192]. Nevertheless, the same reasoning applies, country-specific platforms can potentially bring benefits in representativeness and more closely estimate the country-wide health-related statistics.

The remaining sources, web encyclopedia and online obituaries, are used without a defined geographical restriction, and only English data were considered. Discussion forums and websites are an exception as they were utilized in different language-specific scenarios. Media monitoring systems also work on data from multiple sources and languages.

### Research Question 2: What Is the Aim of the Research Conducted Using These Sources?

With IBSs of health information, the approaches are mostly based on monitoring the internet search and information-sharing behavior; the underlying assumption is that people actively seek and share information on diseases they develop.

In terms of surveillance and outbreak forecasting, estimates of disease activity within a community can be expressed by monitoring the frequency of related internet searches, disease mentions on social and news media, and page views in a web encyclopedia, among others. In addition, these sources also provide complementary information to the ground truth, eg, by targeting sick people who might not go to the hospital. When dealing with outbreak detection, an early and fast response is essential. Traditional surveillance is slower to transmit information across its different channels; therefore, IBSs complement the traditional mechanism when dealing with outbreaks [5,193,194].

Sources that go beyond single keywords pose a challenge as the occurrence of disease mentions does not correspond to the assumption that the text/health report in consideration is referring to the user suffering from the mentioned disease. For example, the microblog “Don’t forget to get your flu shot” is not as valuable as the microblog “I have the flu”; the latter corresponds to a personal health mention that has the potential to more closely correlate with gold standard data. Hence, personal health mention classification is based on the application of classification techniques that aim to filter false-positives, ie, a text containing a disease mention but not stating the user is carrying the disease, from true-positives, ie, a first person mentions of a disease by the affected user [37,72]. This is an important step that has been introduced when dealing with microblogs and online forums, as it has shown improvements for surveillance and outbreak forecasting.

Diagnosis prediction was not a common aim of the analyzed studies as it is difficult to validate owing to the lack of available gold standard data and owing to privacy concerns. The studies by De Choudhury et al [47] and Bodnar et al [64] include a prior user selection process for whom the authors have diagnosis information. In these cases, the source utilized was social media as it provides more contextual information and the potential for sentiment analysis, which is particularly valuable for mental health infodemiology studies. In contrast, the work by Karmen et al [97] targets the diagnosis of a health report (in the form of a forum post) and not the user itself (as not all information for the user is available) utilizing a similar methodology. Yom-tov et al [113], in their study, identify risk markers that correlate with a set of diseases based on the search behavior of assumed affected users.

When considering long-term patients, they also seek the internet for health information but also for online support through the connection with other patients [36,53,86,134]. This corresponds to the task of content analysis and disease characterization. IBSs are not only useful to perform disease surveillance but also to understand the information that is being shared online, which directly relates to public health tasks. The literature also points to the preference of sources for particular user groups, namely, users who seek support groups or connection with other patients and who suffer from chronic illnesses. In this situation, forums and social media, namely, microblogs, provide a suitable medium for the discussion of examination results, symptoms, treatments, and support, offering insights into how diseases are discussed online [36,41,72,86].

### Research Question 3: How Are Internet-Based Sources Applied to Generate Knowledge?

As most of the publications aim to perform disease surveillance and outbreak forecasting, correlation analysis is regularly applied to determine the relationship between the IBSs and ground truth. Surveillance data are also commonly incorporated into surveillance and forecasting using regression models, which can also include autoregression, ie, past values of the ground-truth data. However, these methods make several assumptions regarding the distribution of the data, which might not be correct and overly simplistic.

Studies that utilize multiple sources of external data tend to apply more complex statistical methods which attempt to address the assumptions made by regression and autoregression models in trade of higher complexity.

The techniques mentioned earlier are applied to time-series data that can be obtained from the search query volume, page views from a web encyclopedia, and message/health report frequency in social media. In the latter case, to obtain the frequency, machine learning is commonly used to filter messages that are considered nonrelevant for the disease or medical condition in question. Thus, most of the machine learning approaches focus on social media and are reliant on annotated datasets, ie, a set of messages previously labelled as relevant or nonrelevant, which carry an added cost as this is necessary to train the models, as well as the lack of generalization as the labelled dataset targets a specific disease/medical condition.

Deep learning approaches improve generalization, they are capable of modelling complex nonlinear relationships, and do not impose restrictions on the data, eg, distribution; however, they have much higher complexity and can act as a black box owing to the high number of *tunable* hyperparameters. Furthermore, they require large sets of data that might not be available for diseases with a lower prevalence.

Topic analysis is mostly used for content analysis and it provides added benefits to manual analysis and surveys as it is unsupervised, ie, it does not require human input to perform the analysis. However, the topics identified might not be clearly related to a subject, which can lead to subjective interpretations; furthermore, it also carries high computational costs.

Linguistic analysis, in particular, sentiment analysis, can provide insights regarding negative and positive word use, among others, and how it associates with diseases. For example, this is used for mental health research as the sentiment expressed in words can be fundamental to detect the mental state of a user [82,157]. In the same category, named entity recognition aids in the detection of locations and disease names, among others [144]. Furthermore, the use of lexical and syntactic features and the use of lexicons have shown to improve classification tasks, eg, self-mentions of disease and disease-related categories [81,162].

The use of the epidemiology theory is not common as it can require data that are not available through the use of IBSs owing to its limits in terms of user information (eg, age and location); however, some studies have implemented various epidemiological models [96,121,140,170], as well as epidemiological parameters [34,119].

Rule-based techniques are manually created and are specific to the disease/medical condition studied; hence, they suffer from lack of generalization.

Ranking techniques were only used in 1 of the analyzed papers [195],  and it was used to rank the topics generated from a topic modelling approach, suggesting that these can be used to facilitate the interpretation of the topic analysis results.

### Research Question 4: Which Sources Have Shown a Preference for Studying Communicable and Noncommunicable Diseases, Health Topics, and Mental Health?

The nature of infectious diseases, ie, fast moving and with easier measurable effects, makes these a preferential case study for outbreak detection and surveillance. In tandem, the sources commonly applied for these tasks are search queries and social media, both combined and with other sources. These are the preferred sources as their output can be transformed into time-series data and compared with a gold standard for evaluation. With regard to search queries, a variety has been used to provide the most representative search behavior for the countries and languages targeted. A similar methodology was applied with social media, although mostly restricted to microblogs. Another important task in studying communicable illnesses is to explore what type of information is being shared and when; this is vital to identify the spread of misinformation and to analyze how far-reaching the counteractions are from health care agencies. Hence, content analysis is also performed by mainly utilizing social media as it provides more contextual information than search queries. Forums and news are utilized for the same reason; the higher contextual value allows for more insights into the study of information dissemination.

When discussing NCDs, monitoring and content analysis are the major approaches taken on the papers analyzed. Collecting epidemiological data for NCDs is a labor-intensive process [57,196]; hence, monitoring through digital sources aims primarily to estimate the number of affected individuals, given that official statistics are released with a significant delay [47,57,58,95,136]. To perform such a task, the commonly used sources are social media, search queries, and online obituaries. Content analysis focuses on determining the behavior and characteristics of users who actively mention a disease (eg, through a forum or social media), and the content and dissemination of health information. This is relevant as it allows to explore the information that is spread within these communities, such as personal medical advice. Additionally, past research has shown that online communities can provide a more convenient environment, for some patients, than traditional face-to-face interactions with health providers [36,41,43,82]. For content analysis, the sources applied are social media, forums, and web encyclopedia. As stated earlier, data with greater contextual content could provide more detailed information regarding online behavior and information exchange. With regard to forecasting, Gu et al [95] and Zhang et al [138] mention the task of predicting erythromelalgia-related hospital visits one week ahead and detecting early signals of diabetes, both through the use of search query data.

The research on mental health, medical conditions, and health topics focuses on content analysis; thus, the sources utilized in the analyzed papers refer to social media, in particular, microblogs, and forums.

Overall, the choice of the source of data is significantly related to the health topics, aim of the study, and the data available for evaluation. Infectious diseases have a large incidence variation; hence, they tend to have surveillance data available and most of the approaches focus on surveillance and outbreak forecasting which in itself requires sources that immediately show changes in the online behavior of users. Thus, search queries and microblogs are preferred for an analysis requiring a timely response. Regarding the remaining health topics, the population affected does not fluctuate as is the case with infectious diseases; hence, the focus is on the discussion that occurs online. It is more valuable to determine the information being disseminated, the questions raised online, and the needs of the patients so that the health agencies can cater to this segment of the population.

### Research Question 5: What Are the Common Limitations of Internet-Based Sources?

Most of the studies analyzed report on positive outcomes when utilizing IBSs for public health applications; however, some limitations are frequently cited and only a few authors have given these greater importance. Although recent statistics illustrate the growth in search for health information online [197], internet access is neither equally distributed among countries nor equally penetrating in all regions within a country, which significantly affects the application of IBSs of health information [129,169]. In all the sources, common limitations refer to the lack of representativeness and bias caused by internet penetration and access, and preference to certain user age groups. For example, in the case of Twitter, 62% of its 330 million users are aged between 18 and 49 years [198]; around 53% of American internet users look up information on Wikipedia, with these users being mostly highly educated (69%) and under the age of 30 years (62%) [199]. This type of information elucidates on the potential bias caused by the nongeneralized use of these sources, in particular, when a given age group is more susceptible to a disease (eg, elderly and children).

Another common limitation is related to precision issues caused by the inherent nature of diseases. These tend to share symptoms and treatments that are commonly used as keywords to detect relevant health reports, furthermore, the use of unspecific health-related terminology is also common. The layman language used in IBSs is a challenge, given that most approaches in the field rely on the use of keywords selected from specialized medical vocabulary, as summarized by Dai et al [142]. However, the evolution of learning-based, lexicon-based, and embedding approaches has started to mitigate the language specificity effect.

When ground-truth data are available, some studies question their quality as they can be updated after the initial publication; other mentioned limitations concern the amount of data as well as their timespan, and geographical coverage. Language restriction is also a common limitation of the studies, as most are performed only in English.

In particular to search queries and social media, the lack or limited geographical resolution is also cited as a limitation. Using Google Trends, various studies refer to the lack of transparency on how the search volumes are obtained, especially since forecasting systems based on Google Trends (ie, Google Flu Trends and Google Dengue Trends) have shown significant algorithmic problems that led to their termination [200,201]. The need for costly, manually annotated datasets is a common issue when the goal is to perform classification, and it mainly occurs with social media data.

**Figure 4:**
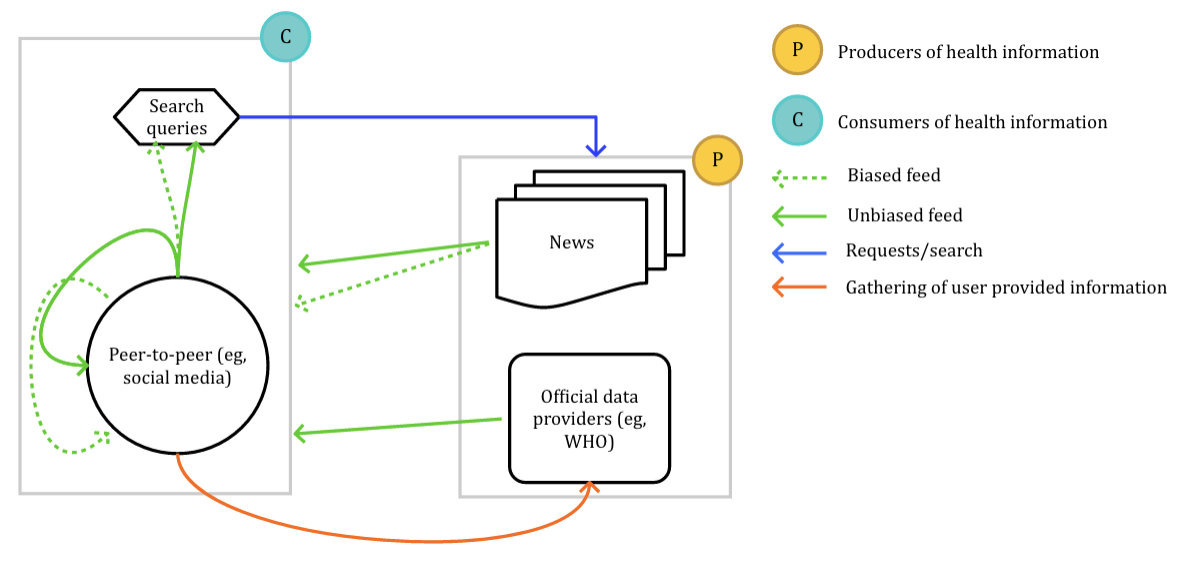
Data sources interaction cycle. WHO: World Health Organization.

In addition, a common limitation to search queries, social media, and web encyclopedia is the effect caused by media events. A media event is an event or activity conducted for media publicity. In this definition, we include examples such as panic-inducing news [109] and celebrities being diagnosed with medical conditions [173]. Media events have shown to significantly affect the reliability of these sources. The results of studies by Yom-tov [109] and Mollema et al [100] demonstrate how these sources show a higher correlation with media events than actual surveillance data. The study by Alicino et al [85] also reveals that the presence of news strongly influences the search volume in locations where an outbreak is not occurring. In light of this, we present an interaction schema in Figure 4; the sources of public health–related data predominantly comprise search engine queries, which target public sources, and peer-to-peer (P2P) social media networks; we can further distinguish primary consumers of health information, eg, members of P2P networks, from producers, eg, biased and unbiased news and unbiased official data providers (eg, governmental sources). News and official data providers deliver biased and unbiased information to the consumers. Consumers receive this information and spread it, affecting their search and share behavior, namely, in search queries, social media, and web encyclopedias.

### Conclusions

IBSs of health information are a valuable proxy to study illnesses affecting the population. Their benefits and applications are far-reaching and continue to evolve as a potential asset to public health. The knowledge gathered from this review suggests that search queries and social media provide useful data to monitor infectious diseases. With regard to studying chronic illnesses, discussion forums and social media are preferred. 

The methods used to select relevant keywords or messages target specific illnesses, thus requiring constant updates to reflect the population’s changing search behavior as well as emerging trends. Here, we identify the first research gap; disease outbreaks outside of the targeted disease names will not be identified, and new terminologies crucial for the detection of previously targeted diseases will be missed. Future approaches must focus on the ever-changing nature of diseases. For example, new related keywords could be identified through services such as Google Trends’ *related topics*. To identify emerging illnesses, more emphasis must be given to the structure and syntax of messages describing a first-person mention of a disease, as this could be applicable to other illnesses. 

The strong susceptibility to media events and the absence of approaches dealing with this issue constitute the second research gap. As shown in Figure 4, the interactions between the different sources and the type of information (biased and unbiased) reach the consumers and affect their search and share behavior, namely, in search queries, social media, and web encyclopedias. Such an effect must be mitigated to ensure improved reliability when utilizing IBSs. 

The third research gap relates to the absence of consistent training and test periods, which impedes the appropriate comparison among the different methodologies. To address this, we suggest the creation of standard datasets, allowing to quantify the improvements of distinct methodologies. We also found a significant lack of interaction with public health officials, which would be the entities receiving the information from these models.

As a final recommendation, we suggest the use of an alternative strategy to better harness the information provided by IBSs. Namely, a proactive approach where the users are asked to report on their health state requesting the user to anonymously publish this information while avoiding the inclination to only publish positive messages. Such implementations can potentially make IBSs more comprehensible and a more valuable asset for disease monitoring.

### Systematic Literature Review Limitations

This study makes use of eight databases, aiming to achieve a high coverage of the scientific literature. However, these databases do not guarantee full coverage and, hence, the inclusion of all relevant publications in our systematic methodology. In addition, we only considered articles in English as it is the predominant language of the scientific literature; thus, some contributions were potentially missed. The publication period is restricted to the last 6 years to allow for a focus on recent trends; earlier studies were referenced in the Introduction; however, not in an exhaustive way. We included a variety of keywords for the literature search although we understand that these might not cover all relevant publications.

Given that the authors followed a rigorous and systematic methodology when including and excluding publications for this literature review, selection bias was minimized. However, we cannot guarantee the absence of a bias when qualitatively presenting the findings; some categories and articles might be over- or under-represented.

## Appendix

Multimedia Appendix 1 Supporting information for the systematic literature review (keywords, inclusion and exclusion criteria, quality assessment, data extraction checklist, literature synthesis/summary).

## Abbreviations

IBS: internet-based source
ILI: influenza-like illness
NCD: noncommunicable disease
P2P: peer-to-peer
RQ: research question
SFI: Science Foundation Ireland
