# Supplementary material for: The Application of Internet-Based Sources for Public Health Surveillance (Infoveillance): Systematic Review
Source: J Med Internet Res. 2020 Mar 13;22(3):e13680. doi: 10.2196/13680 (PMC7101503; doi:10.2196/13680)
Supplement: Multimedia Appendix 1 [file jmir_v22i3e13680_app1.docx]

**Supplementary Information**

*Table 1 - Keywords utilised for the literature search. The search applied “AND” and “OR” Boolean functions to generate keyword combinations.*

| disease AND | surveillance OR outbreak OR monitoring |
| --- | --- |
| disease AND web AND 2.0 AND |  |
| digital AND disease AND |  |
| public AND health AND |  |
| syndromic AND surveillance AND | outbreak OR monitoring |
| event AND based AND surveillance AND | syndromic OR disease |
| syndromic AND monitoring | |
| syndromic AND outbreak | |
| forecasting AND outbreak | |
| digital AND surveillance | |
| digital AND outbreak | |
| disease AND forecasting | |
| public AND health AND web AND 2.0 | |
| event AND based AND surveillance AND syndromic | |
| event AND based AND surveillance AND syndromic | |
| event AND based AND surveillance | |
| infoveillance | |
| infodemiology | |
| digital epidemiology | |

*Table 2 - Criteria for inclusion or exclusion of articles according to a modified PICOTS. The parameter “Patients” was adapted to “Source”, “Setting” to “Study type”, and we included “Language”.*

| **Parameter** | **Inclusion Criteria** | **Exclusion Criteria** |
| --- | --- | --- |
| Source | Utilises or explores an Internet-based source of health information | Source of health information not originated from informal and Internet-based sources (eg, electronic health records) |
| Intervention | Utilises or explores an Internet-based source of health information for public health applications while focusing on a medical condition and/or illness | Only describes the development of an algorithm, model, or tool. Focus on illicit or pharmaceutical drug use and/or abuse |
| Comparator | None | None |
| Outcomes | Tests and states potential implementations in public health, focusing on a medical condition and/or illness | Development of an algorithm, model, or tool |
| Timeframe | 2012-2018 | Before 2012 and after 2018 |
| Study Type | Research Articles and Conferences Proceedings | Brief Reports, Letter to Editors, Communications, Data Articles, Viewpoints, Clinical Studies, Prospectives, Supplements, Tutorials, Reviews |
| Language | English | Non-English |

QC 1 Is there is a clear statement of the aim of the research?

QC 2 Is the study is put into context of other studies and research?

QC 3 Are system or algorithmic design decisions justiﬁed?

QC 4 Is the test data set reproducible?

QC 5 Is the study algorithm reproducible?

QC 6 Is the experimental procedure throughly explained and reproducible?

QC 7 Is it clearly stated in the study which other algorithms the study’s algorithm(s) have

*Table 3 – Criteria utilised for the quality assessment based on Kofod-Petersen.*

| **Quality Criteria** |
| --- |
| Is there is a clear statement of the aim of the research? |
| Is the study is put into context of other studies and research? |
| Are system or algorithmic design decisions justified? |
| Is the test data set reproducible? |
| Is the study algorithm reproducible? |
| Is the experimental procedure thoroughly explained and reproducible? |
| Is it clearly stated in the study which other algorithms the study’s algorithm(s) have been compared with? |
| Are the performance metrics used in the study explained and justified? |
| Are the test results thoroughly analysed? |
| Does the test evidence support the findings presented? |

*Table 4 – Checklist for the information extracted from the literature. Each checklist item was further classified accordingly to the available options.*

| **Checklist Item** | **Classification** |
| --- | --- |
| Type | Conference or Journal |
| Goal/Objective | Content analysis, Personal health mention classification, Diagnosis prediction, Disease characterisation, Outbreak forecasting, Surveillance |
| Disease/Medical condition/Health topics | Infectious (Zika virus disease, West Nile virus disease, Tuberculosis, Syphilis, Scarlet fever, Pneumonia, Plague, Pertussis, Mumps, Middle East Respiratory Syndrome, Meningitis, Malaria, Lyme, Leptospirosis, Infectious intestinal diseases, HIV-AIDS, Hepatitis, Hand-foot-mouth disease, Gonorrhoea, Influenza-like-illnesses, Ebola virus disease, Dengue fever, Conjunctivitis, Chikungunya virus disease, Chickenpox, Chlamydia), Chronic (Lupus, Multiple Sclerosis, Heart diseases, Fibromyalgia, Diabetes, Chron disease, Cancer, Autism, Asthma, Allergies, Erythromelalgia), Mental health, Health topics, Medical conditions (Pregnancy-related, Whiplash, Obesity, Kidney stones, Cardiac arrest) |
| Internet-based Data Source | Search queries (Google Trends, Google Dengue Trends, Google Flu Trends, Baidu, Websök, Vårdguiden, Naver), Web encyclopedia (Wikipedia), Websites, News, Social Media (Twitter, Facebook, YouTube, Daum, Weibo), Forums, Online obituaries, Media monitoring systems (MedIsys, HealthMap) |
| Data Collection  (external sources to the Internet-based) | Socioeconomic statics, Climate and temperature statistics, Governmental and laboratory surveillance statistics, Demographic or population statistics, Hospital and emergency department visits, Scientific search engines, Pharmaceutical sales, Health records, Telephone triage |
| Study Design | Topic analysis, Regression models, Machine Learning, Deep Learning, Correlation analysis, Statistical models, Ranking techniques, Epidemiology theory, Manual analysis, Rule-based techniques, Linguistic analysis |
| Findings | None |
| Limitations | None |

*Table 5 – Summary of the literature reviewed according to the extraction criteria. “S. Category”, “D. Category”, “External IBS”, and “Ref” represent “Source Category”, “Disease Category”, “External to Internet-based Sources”, and “Reference”. The abbreviation representation can be found in the manuscript. In the column “Type”, “J” and “C” represents “Journal” and “Conference”. The remaining abbreviations correspond to Climate or Temperature Statistics (CTS), Demographic or Population Statistics (DS), Environmental Statistics (ES), Flu Near You (FNY), Google Dengue Trends (GDT), Google Flu Trends (GFT), Governmental or Laboratory Surveillance Statistics (GSS), Google Trends (GT), Hand-Foot-Mouth Disease (HFMD), Hospital or Emergency department visits (Hospital visits), Health Records (HR), Infectious Intestinal Diseases (IID), Media Monitoring System (MMS), Multiple Sclerosis (MS), Non-Applicable (NA), Personal Health Mention Classification (PHMC), Pregnancy-Related Medical Conditions (PRC), Rule-Based Techniques (RBT), Socioeconomic Statistics (SES), Social Media (SM), Search Queries (SQ), West Nile Virus (WNV).*

| ***Year*** | ***Type*** | ***Source*** | ***S. Category*** | ***Disease*** | ***D. Category*** | ***External IBS*** | ***Goal*** | ***Study Design*** | ***Ref*** |
| --- | --- | --- | --- | --- | --- | --- | --- | --- | --- |
| 2012 | C | Twitter & MedISys | SM & MMS | IID | Infectious | NA | Outbreak Forecasting | Topic Analysis & Ranking Techniques | [195] |
| 2012 | J | Twitter & HealthMap | SM & MMS | Cholera | Infectious | GSS | Surveillance | Correlation Analysis & Epidemiology Theory | [34] |
| 2012 | J | GFT | SQ | ILI | Infectious | Hospital visits | Surveillance | Correlation Analysis | [35] |
| 2012 | J | Forums | Forums | PRC | Medical conditions | NA | Content Analysis | Surveys | [36] |
| 2012 | C | Twitter | SM | ILI | Infectious | NA | PHMC | Machine Learning | [37] |
| 2012 | J | Forums | Forums | Mental Health | Mental Health | NA | Content Analysis | Manual Analysis | [27] |
| 2012 | J | GFT | SQ | ILI | Infectious | Pharmaceutical sales & GSS | Surveillance | Correlation Analysis | [38] |
| 2012 | J | GFT | SQ | ILI | Infectious | GSS | Outbreak Forecasting | Statistical Models | [39] |
| 2012 | J | GT | SQ | Scarlet fever | Infectious | GSS | Surveillance & Outbreak Forecasting | Regression Models | [40] |
| 2012 | J | Twitter | SM | Cancer | Chronic | NA | Content Analysis | Manual Analysis | [41] |
| 2012 | J | News & Twitter & Facebook & Hyves & StudiVZ & Websites & Wikipedia | News & SM & Websites & Web Encyclopedia | IID | Infectious | NA | Content Analysis | Manual Analysis & Surveys | [42] |
| 2012 | J | Twitter | SM | Cardiac arrest | Medical conditions | NA | Content Analysis | Manual Analysis | [43] |
| 2013 | J | GT | SQ | MS | Chronic | GSS | Surveillance | Regression Models | [44] |
| 2013 | J | Twitter | SM | ILI | Infectious | GSS | Surveillance | Correlation Analysis | [45] |
| 2013 | J | GT | SQ | ILI | Infectious | GSS | Surveillance | Correlation Analysis | [46] |
| 2013 | C | Twitter | SM | Mental Health | Mental Health | GSS | Diagnosis Prediction | Machine Learning | [47] |
| 2013 | J | GFT | SQ | ILI | Infectious | Hospital visits & CTS | Outbreak Forecasting | Regression Models | [48] |
| 2013 | J | Twitter | SM | ILI | Infectious | GSS | Surveillance | Correlation Analysis | [49] |
| 2013 | J | Twitter | SM | Obesity | Medical conditions | NA | Content Analysis | Topic Analysis | [50] |
| 2013 | J | GT | SQ | ILI | Infectious | GSS | Surveillance | Correlation Analysis | [51] |
| 2013 | J | Twitter | SM | ILI | Infectious | GSS | Outbreak Forecasting | Regression Models | [52] |
| 2013 | J | Forums | Forums | Multiple | Chronic | NA | Content Analysis | Topic Analysis | [53] |
| 2013 | J | Twitter | SM | Multiple | Infectious | GSS | Content Analysis & Surveillance & Disease Characterization | Correlation Analysis | [54] |
| 2013 | J | GFT | SQ | ILI | Infectious | GSS & Hospital visits | Surveillance & Outbreak Forecasting | Correlation Analysis | [55] |
| 2013 | C | Wikipedia & Twitter | Web Encyclopedia & SM | Multiple | Multiple | NA | Surveillance | Topic Analysis | [56] |
| 2013 | J | GT | SQ | Kidney Stones | Medical conditions | CTS & GSS | Surveillance | Correlation Analysis | [57] |
| 2013 | J | Naver | SQ | Mental Health | Medical conditions | CTS & SES & GSS | Surveillance | Regression Models | [58] |
| 2013 | J | Yandex & GT | SQ | HIV-AIDS | Infectious | GSS | Surveillance & Content Analysis | Correlation Analysis & Statistical Models | [59] |
| 2013 | C | GT & Wikipedia | SQ & Web Encyclopedia | Multiple | Infectious | GSS | Outbreak Forecasting | Statistical Models | [60] |
| 2014 | J | Vårdguiden | SQ | IID | Infectious | GSS & Pharmaceutical sales & Telephone triage | Surveillance | Regression Models | [61] |
| 2014 | J | GFT | SQ | ILI | Infectious | GSS & Hospital visits | Outbreak Forecasting | Regression Models | [62] |
| 2014 | J | Twitter | SM | ILI | Infectious | GSS & Hospital visits | Surveillance | Machine Learning | [63] |
| 2014 | C | Twitter | SM | ILI | Infectious | HR | Diagnosis Prediction | Machine Learning | [64] |
| 2014 | C | Twitter | SM | ILI | Infectious | GSS | Surveillance & Outbreak Forecasting | Topic Analysis | [65] |
| 2014 | C | Twitter | SM | Health topics | Health topics | GSS | Content Analysis | Regression Models | [66] |
| 2014 | J | YouTube | SM | WNV | Infectious | NA | Content Analysis | Manual Analysis | [67] |
| 2014 | J | Websök | SQ | IID | Infectious | GSS | Surveillance & Outbreak Forecasting | Correlation Analysis | [68] |
| 2014 | J | Wikipedia | Web Encyclopedia | Multiple | Infectious | GSS | Surveillance & Outbreak Forecasting | Regression Models | [69] |
| 2014 | J | GDT | SQ | Dengue | Infectious | GSS & CTS & SES | Surveillance | Correlation Analysis | [70] |
| 2014 | J | Baidu & Weibo | SQ & SM | ILI | Infectious | GSS | Surveillance & Content Analysis | Correlation Analysis | [71] |
| 2014 | J | Twitter & News | SM & News | ILI | Infectious | GSS | Surveillance & Outbreak Forecasting | RBT | [21] |
| 2014 | J | Forums | Forums | HIV-AIDS | Infectious | GSS | PHMC | Machine Learning | [72] |
| 2014 | J | Wikipedia & GFT | Web Encyclopedia & SQ | ILI | Infectious | GSS | Surveillance | Statistical Models | [73] |
| 2014 | J | GT | SQ | Infectious | Infectious | GSS | Surveillance | Correlation Analysis | [74] |
| 2014 | J | Twitter | SM | ILI | Infectious | GSS & Hospital visits | Outbreak Forecasting | Statistical & Regression Models | [75] |
| 2014 | J | Daum | SQ | ILI | Infectious | GSS | Surveillance | Regression Models | [76] |
| 2014 | J | Twitter | SM | Health topics | Health topics | GSS | Content Analysis & Surveillance & Disease Characterization | Topic Analysis | [77] |
| 2014 | J | Twitter | SM | Multiple | Multiple | NA | PHMC & Content Analysis | RBT & Machine Learning | [78] |
| 2014 | J | Twitter & Sapo | SM & SQ | ILI | Infectious | GSS | Surveillance | Regression Models & Machine Learning | [79] |
| 2014 | J | GFT | SQ | ILI | Infectious | GSS & Hospital visits | Surveillance | Correlation Analysis | [80] |
| 2014 | J | Twitter | SM | Multiple | Multiple | GSS | Surveillance | RBT & Correlation Analysis | [81] |
| 2014 | C | Twitter | SM | Mental Health | Mental Health | NA | Content Analysis | Linguistic Analysis | [82] |
| 2014 | J | Bing & Twitter | SQ & SM | Health topics | Health topics | NA | Outbreak Forecasting | Statistical Models | [83] |
| 2014 | J | Twitter | SM | HIV-AIDS | Infectious | GSS | Surveillance | Regression Models | [84] |
| 2015 | J | GT | SQ | Ebola | Infectious | GSS | Surveillance | Regression Models | [85] |
| 2015 | J | Forums | Forums | PRC | Medical conditions | NA | Content Analysis | Manual Analysis | [86] |
| 2015 | J | GFT & Twitter | SQ & SM | ILI | Infectious | GSS & Hospital visits | Surveillance | Regression Models | [87] |
| 2015 | J | News | News | Ebola | Infectious | GSS | Content Analysis | Manual Analysis | [88] |
| 2015 | J | Weibo | SM | ILI | Infectious | GSS | Outbreak Forecasting | Machine Learning | [89] |
| 2015 | J | GFT | SQ | ILI | Infectious | GSS | Surveillance & Outbreak Forecasting | Regression Models | [90] |
| 2015 | J | GT | SQ | ILI | Infectious | GSS | Surveillance | Regression Models | [91] |
| 2015 | J | Twitter | SM | Heart diseases | Medical conditions | GSS & SES | Surveillance & Content Analysis | Correlation Analysis & Linguistic Analysis | [92] |
| 2015 | J | Weibo | SM | Infectious | Infectious | NA | Content Analysis | Manual Analysis | [93] |
| 2015 | J | Twitter | SM | Allergies | Chronic | GSS | Surveillance | Correlation Analysis | [94] |
| 2015 | J | Facebook | SM | Multiple | Multiple | DS | Surveillance | Regression Models | [25] |
| 2015 | J | Baidu | SQ | Erythromelalgia | Chronic | GSS & CTS | Outbreak Forecasting | Regression Models | [95] |
| 2015 | J | Wikipedia | Web Encyclopedia | ILI | Infectious | GSS | Outbreak Forecasting | Statistical Models & Epidemiology Theory | [96] |
| 2015 | J | Forums | Forums | Mental Health | Mental Health | NA | Diagnosis Prediction | Linguistic Analysis & RBT | [97] |
| 2015 | J | Online obituaries | Online obituaries | Multiple | Multiple | GSS | Surveillance | Machine Learning & RBT | [98] |
| 2015 | C | Twitter | SM | Allergies | Chronic | GSS & CTS | Surveillance | Machine Learning | [99] |
| 2015 | J | Twitter & News & Forums & Facebook | SM & News & Forums | Measles | Infectious | GSS | Content Analysis | Manual Analysis | [100] |
| 2015 | J | Twitter | SM | Ebola | Infectious | NA | Surveillance & Content Analysis | Machine Learning | [101] |
| 2015 | J | Twitter | SM | Health topics | Multiple | NA | Surveillance | Topic Analysis | [102] |
| 2015 | J | YouTube | SM | Ebola | Infectious | NA | Content Analysis | Manual Analysis | [103] |
| 2015 | J | GT | SQ | Pertussis | Infectious | GSS | Surveillance | Regression Models | [104] |
| 2015 | J | Twitter & GT | SM & SQ | Asthma | Chronic | Hospital visits & ES | Surveillance | Deep Learning | [105] |
| 2015 | C | Twitter & Facebook | SM | Chron | Chronic | NA | Content Analysis | Manual Analysis | [29] |
| 2015 | J | Twitter & GFT & GT | SM & SQ | ILI | Infectious | GSS & HR & FNY | Surveillance & Outbreak Forecasting | Machine Learning & Regression Models | [106] |
| 2015 | J | Twitter | SM | Mental Health | Medical conditions | NA | Content Analysis | Surveys & Regression Models | [107] |
| 2015 | C | Twitter | SM | HIV-AIDS | Infectious | NA | Disease Characterization | RBT | [108] |
| 2015 | C | Bing & Twitter & News | SQ & SM & News | Ebola | Infectious | GSS | Surveillance | Correlation Analysis | [109] |
| 2015 | J | GT | SQ | Mental Health | Medical conditions | Hospital visits | Surveillance & Outbreak Forecasting | Correlation Analysis | [110] |
| 2015 | J | GT | SQ | ILI | Mental Health | GSS | Outbreak Forecasting | Regression Models | [111] |
| 2015 | J | Twitter | SM | Health topics | Health topics | NA | PHMC | Machine Learning | [112] |
| 2015 | J | Bing & Wikipedia | SQ & Web Encyclopedia | Health topics | Health topics | NA | Diagnosis Prediction | Machine Learning | [113] |
| 2015 | J | Baidu | SQ | ILI | Infectious | GSS | Surveillance | Regression Models | [114] |
| 2015 | C | Twitter & GT | SQ & SM | Multiple | Multiple | NA | Content Analysis & Surveillance | Correlation Analysis | [115] |
| 2015 | J | Weibo | SM | ILI | Infectious | GSS | Surveillance & Content Analysis | Correlation Analysis | [116] |
| 2015 | J | Wikipedia | Web Encyclopedia | Ebola | Infectious | GSS | Surveillance | Linguistic Analysis | [144] |
| 2015 | J | Twitter | SM | ILI | Infectious | GSS | PHM & Surveillance | Machine Learning | [117] |
| 2016 | J | Twitter | SM | ILI | Infectious | GSS | Surveillance | Machine Learning & Correlation Analysis | [118] |
| 2016 | J | GT | SQ | Chicken pox | Infectious | GSS | Surveillance & Outbreak Forecasting | Epidemiology theory & Correlation analysis | [119] |
| 2016 | C | Twitter | SM | ILI | Infectious | GSS | Surveillance | Machine Learning | [120] |
| 2016 | J | Twitter | SM | ILI | Infectious | GSS | Surveillance | Epidemiology theory & Topic Analysis | [121] |
| 2016 | J | Twitter & GT | SM & SQ | Eye disease | Infectious | HR | Surveillance | Correlation Analysis | [122] |
| 2016 | C | HealthMap | SMM | Multiple | Multiple | NA | Disease Characterization | Deep Learning | [123] |
| 2016 | J | Twitter | SM | Multiple | Multiple | NA | PHM & Content Analysis | Machine Learning & Correlation Analysis | [124] |
| 2016 | J | GFT | SQ | ILI | Infectious | Hospital visits & SES & DS | Surveillance | Correlation Analysis | [125] |
| 2016 | J | GT | SQ | Multiple | Multiple | GSS | Surveillance | Correlation Analysis | [126] |
| 2016 | J | HealthMap & GT | SMM & SQ | Zika | Infectious | GSS | Surveillance | Statistical Models | [127] |
| 2016 | J | GFT | SQ | ILI | Infectious | GSS & Hospital visits | Surveillance | Correlation Analysis | [128] |
| 2016 | J | GFT | SQ | ILI | Infectious | GSS | Outbreak Forecasting | Statistical Models | [129] |
| 2016 | J | GFT | SQ | ILI | Infectious | GSS | Surveillance | Correlation Analysis | [130] |
| 2016 | J | Forums | Forums | Health topics | Health topics | NA | Content Analysis | Manual Analysis | [131] |
| 2016 | J | Twitter | SM | Measles | Infectious | NA | Content Analysis | RBT & Manual Analysis | [132] |
| 2016 | J | Twitter & Wikipedia & GT | SM & SQ & Web Encyclopedia | ILI | Infectious | GSS | Surveillance | Statistical Models | [133] |
| 2016 | J | Naver | SQ | ILI | Infectious | GSS | Surveillance | Correlation Analysis | [134] |
| 2016 | C | Twitter | SM | Health topics | Health topics | NA | Content Analysis | Topic Analysis | [135] |
| 2016 | J | Online obituaries | Online obituaries | Cancer | Chronic | GSS | Surveillance | Correlation Analysis | [136] |
| 2016 | J | Naver & Daum | SQ & SM | ILI | Infectious | GSS | Outbreak Forecasting | Machine Learning | [137] |
| 2016 | C | Twitter | SM | Asthma | Chronic | GSS & Hospital visits | Surveillance & Outbreak Forecasting | Machine Learning | [138] |
| 2016 | C | Twitter | SM | IID | Infectious | GSS | Surveillance | Deep Learning | [139] |
| 2017 | C | Twitter | SM | Dengue | Infectious | GSS | Outbreak Forecasting | Epidemiology theory & Statistical Models | [140] |
| 2017 | J | News & GT | SQ & News | HIV-AIDS | Infectious | NA | Content Analysis | Correlation Analysis | [141] |
| 2017 | C | Twitter | SM | ILI | Infectious | NA | Disease Characterization | Deep Learning | [142] |
| 2017 | J | Baidu | SQ | HFMD | Infectious | GSS & CTS | Outbreak Forecasting | Regression Models | [143] |
| 2017 | J | Twitter | SM | MERS | Infectious | NA | Content Analysis | Manual Analysis & Correlation Analysis | [145] |
| 2017 | J | HealthMap & News | SMM & News | Multiple | Multiple | GSS | Surveillance & Outbreak Forecasting | Topic Analysis & Regression Models | [146] |
| 2017 | J | Baidu | SQ | ILI | Infectious | GSS | Surveillance | Statistical Models | [147] |
| 2017 | J | Twitter | SM | Fibromyalgia | Chronic | NA | Content Analysis | Correlation Analysis & Linguistic Analysis | [148] |
| 2017 | J | Twitter | SM | ILI | Infectious | Hospital visits | Surveillance & Outbreak Forecasting | Correlation Analysis & Regression Models & Machine Learning | [149] |
| 2017 | J | GFT | SQ | ILI | Infectious | GSS | Surveillance | Regression Models | [150] |
| 2017 | C | Twitter & GT | SM & SQ | ILI | Infectious | GSS | Surveillance | Deep Learning & Regression Models | [151] |
| 2017 | C | Twitter & GFT | SM & SQ | ILI | Infectious | GSS | Outbreak Forecasting | Deep Learning & Correlation Analysis | [152] |
| 2017 | J | Baidu | SQ | Dengue | Infectious | GSS & CTS | Outbreak Forecasting | Statistical Models & Correlation Analysis | [153] |
| 2017 | J | Baidu | SQ | IID | Infectious | GSS | Outbreak Forecasting | Statistical Models & Correlation Analysis | [154] |
| 2017 | J | GT & HealthMap & Twitter | SM & SQ & SMM | Zika | Infectious | GSS | Outbreak Forecasting | Statistical Models & Correlation Analysis | [155] |
| 2017 | J | GT | SQ | Zika | Infectious | GSS | Outbreak Forecasting | Correlation Analysis & Regression Models & Machine Learning | [156] |
| 2017 | J | Twitter | SM | Mental Health | Mental Health | NA | Content Analysis | Linguistic Analysis | [157] |
| 2017 | J | GT | SQ | Whiplash | Medical conditions | NA | Content Analysis | Correlation Analysis | [158] |
| 2017 | C | Wikipedia | Web Encyclopedia | Multiple | Infectious | GSS | Surveillance | Regression Models & Correlation Analysis | [159] |
| 2017 | C | Forums | Forums | Diabetes | Chronic | NA | Content Analysis | Machine Learning | [160] |
| 2017 | J | Twitter & Facebook | SM | Chron | Chronic | NA | Content Analysis | Topic Analysis & Linguistic Analysis | [161] |
| 2017 | C | Twitter | SM | Multiple | Infectious | NA | Surveillance | Machine Learning | [162] |
| 2017 | J | GT | SQ | ILI | Infectious | GSS | Surveillance | Regression Models | [163] |
| 2017 | J | Wikipedia & GT | Web Encyclopedia & SQ | Lupus | Chronic | Scientiﬁc search tools | Content Analysis | Manual Analysis & Correlation Analysis | [30] |
| 2017 | J | GDT | SQ | Dengue | Infectious | GSS | Surveillance | Correlation Analysis | [164] |
| 2017 | J | GT | SQ | Zika | Infectious | GSS | Outbreak Forecasting | Regression Models & Correlation Analysis | [165] |
| 2017 | J | GT | SQ | Diabetes | Chronic | GSS | Surveillance | Regression Models & Correlation Analysis & Statistical Analysis | [166] |
| 2017 | J | Twitter & GT & Wikipedia | Web Encyclopedia & SM & SQ | Dengue | Infectious | GSS & DS & SES | Surveillance & Outbreak Forecasting | Regression Models & Correlation Analysis | [167] |
| 2017 | J | GT | SQ | ILI | Infectious | GSS | Outbreak forecasting | Deep Learning & Statistical Models & Regression Models | [168] |
| 2017 | J | GT | SQ | ILI | Infectious | GSS & HR | Surveillance & Outbreak Forecasting | Regression Models | [32] |
| 2017 | C | Twitter | SM | ILI | Infectious | GSS | Surveillance | Regression Models & Machine Learning | [169] |
| 2017 | C | Twitter | SM | ILI | Infectious | GSS | Outbreak Forecasting | Epidemiology theory & Statistical Models & Correlation Analysis | [170] |
| 2017 | J | GT | SQ | Pertussis | Infectious | GSS | Surveillance & Outbreak Forecasting | Regression Models & Correlation Analysis | [171] |
| 2018 | J | Twitter & News | SM & News | Multiple | Multiple | NA | Content Analysis | Linguistic Analysis & Manual Analysis | [172] |
| 2018 | J | Wikipedia | Web Encyclopedia | MS | Chronic | NA | Content Analysis | Manual Analysis | [173] |
| 2018 | C | GT | SQ | ILI | Infectious | GSS & Hospital visits | Outbreak Forecasting | Correlation Analysis & Regression Models | [174] |
| 2018 | J | Weibo | SM | ILI | Infectious | NA | Content Analysis | Manual Analysis | [175] |
| 2018 | J | GT | SQ | Pertussis | Infectious | GSS | Surveillance & Outbreak Forecasting | Correlation Analysis | [176] |
| 2018 | J | GDT | SQ | Dengue | Infectious | GSS | Surveillance & Content Analysis | Correlation Analysis & Linguistic Analysis | [177] |
| 2018 | J | Twitter | SM | ILI | Infectious | NA | Content Analysis & PHMC | Machine Learning & Topic Analysis | [178] |
| 2018 | C | Twitter | SM | Multiple | Multiple | NA | PHMC | Linguistic Analysis & Deep Learning | [179] |
| 2018 | C | Twitter | SM | Dengue | Infectious | GSS | Surveillance & PHMC | Deep Learning & Correlation Analysis | [180] |
| 2018 | J | GT | SQ | Multiple | Multiple | GSS | Surveillance & Content Analysis | Correlation Analysis & Regression Models | [181] |
| 2018 | J | Baidu | SQ | HIV-AIDS | Infectious | GSS | Outbreak Forecasting | Deep Learning | [182] |
| 2018 | J | GT | SQ | Cancer | Chronic | GSS | Content Analysis & Surveillance | Correlation Analysis & Regression Models | [183] |
| 2018 | J | GT | SQ | IID | Infectious | GSS & Hospital visits | Surveillance & Content Analysis | Correlation Analysis & Regression Models | [184] |
| 2018 | J | GT | SQ | Multiple | Infectious | GSS | Outbreak Forecasting | Correlation Analysis & Regression Models | [185] |
| 2018 | J | Twitter | SM | ILI | Infectious | GSS | Surveillance & PHMC | Machine Learning & Regression Models | [186] |
| 2018 | J | GT | SQ | Syphilis | Infectious | GSS | Outbreak Forecasting & Surveillance | Correlation Analysis & Statistical Models | [187] |
| 2018 | C | GT | SQ | ILI | Infectious | GSS | Surveillance | Regression Models & Statistical Models | [188] |

*Table 6 – Summary of the literature reviewed findings and limitations. “Outcome” is expressed in Positive (P), Negative (N) and the remaining columns with Yes or No. The columns to the right correspond to the limitations stated in the literature. Non Applicable (NA) is used in the Findings when the aim of the study is Content Analysis. NA is also used in the Limitations when the authors do not state any limitation to the study.*

| ***Findings*** | | ***Limitations*** | | | | | | | | | |
| --- | --- | --- | --- | --- | --- | --- | --- | --- | --- | --- | --- |
| ***Outcome*** | ***Prediction Capacity*** | ***Ground Truth*** | ***Representation*** | ***Media Effect*** | ***Bias*** | ***Methodology*** | ***Data size*** | ***Timeframe*** | ***Geography*** | ***Language*** | ***Ref*** |
| P | Y |  |  |  |  | Y |  |  |  |  | [195] |
| P |  |  | Y | Y |  |  |  |  |  |  | [34] |
| P |  |  | Y |  |  |  |  | Y | Y |  | [35] |
| NA |  |  | Y |  |  | Y |  |  |  |  | [36] |
| P |  |  |  |  |  | Y |  |  |  |  | [37] |
| NA |  |  | Y |  |  |  |  |  |  |  | [27] |
| P |  |  | Y |  |  |  |  |  | Y |  | [38] |
| P | Y |  | Y |  |  |  |  |  |  |  | [39] |
| P | Y | Y | Y |  |  |  |  |  |  |  | [40] |
| NA |  |  | Y |  |  |  |  |  |  |  | [41] |
| NA |  |  | Y |  |  | Y |  | Y |  |  | [42] |
| NA |  | Y | Y |  |  | Y |  | Y |  |  | [43] |
| P |  |  |  |  |  | Y |  |  |  |  | [44] |
| P |  |  | Y |  |  |  |  |  |  |  | [45] |
| P | N |  | Y |  |  | Y |  |  |  |  | [46] |
| P |  |  | Y |  |  |  |  |  |  |  | [47] |
| P |  |  | Y |  |  |  |  | Y | Y |  | [48] |
| P |  |  | Y |  |  | Y |  |  | Y |  | [49] |
| NA |  |  | Y |  | Y | Y |  |  |  |  | [50] |
| P |  |  |  | Y |  | Y | Y |  |  |  | [51] |
| P | Y |  |  | Y |  |  |  | Y | Y |  | [52] |
| NA |  |  |  |  |  | Y |  |  |  |  | [53] |
| P&N | Y&N | Y |  |  |  | Y |  |  |  |  | [54] |
| N | N | Y | Y |  |  |  |  |  |  |  | [55] |
| P |  |  |  |  | Y | Y |  |  | Y |  | [56] |
| P |  |  | Y | Y |  | Y |  |  |  |  | [57] |
| P |  |  |  |  |  | Y |  |  |  |  | [58] |
| P |  | Y | Y |  |  |  |  |  |  |  | [59] |
| P | Y |  |  | Y |  |  |  |  |  |  | [60] |
| P |  |  | Y |  |  |  |  |  |  |  | [61] |
| P | Y |  |  |  |  | Y |  |  |  |  | [62] |
| P |  |  |  |  |  |  |  |  | Y |  | [63] |
| P |  | NA | | | | | | | | | [64] |
| P | Y |  |  |  |  | Y |  |  |  |  | [65] |
| P |  | NA | | | | | | | | | [66] |
| NA |  | NA | | | | | | | | | [67] |
| P | Y |  | Y |  |  |  |  |  |  |  | [68] |
| P | Y |  |  | Y |  | Y |  |  |  |  | [69] |
| P |  | NA | | | | | | | | | [70] |
| P |  |  |  |  |  | Y |  | Y |  | Y | [71] |
| P | Y |  |  |  |  | Y |  |  |  |  | [21] |
| P |  |  |  |  |  | Y |  |  |  | Y | [72] |
| P |  |  |  |  |  |  |  |  |  | Y | [73] |
| P |  |  | Y |  |  |  |  | Y |  | Y | [74] |
| P | Y | Y |  |  |  |  |  |  |  |  | [75] |
| P |  |  | Y |  |  |  |  |  |  |  | [76] |
| P |  |  | Y |  |  |  |  |  |  |  | [77] |
| P |  |  |  |  |  | Y |  |  |  | Y | [78] |
| P |  |  | Y |  |  |  |  |  |  |  | [79] |
| P |  |  | Y |  |  |  |  | Y | Y |  | [80] |
| P |  | Y | Y |  |  | Y |  |  |  |  | [81] |
| NA |  | NA | | | | | | | | | [82] |
| P | Y | Y |  |  |  | Y |  |  |  |  | [83] |
| P |  | Y |  |  |  | Y |  |  | Y |  | [84] |
| N |  |  | Y |  |  |  |  |  |  |  | [85] |
| NA |  |  | Y |  |  |  |  |  |  |  | [86] |
| P |  |  |  |  |  |  |  |  | Y |  | [87] |
| NA |  |  | Y |  |  | Y |  |  |  |  | [88] |
| P | Y |  | Y |  |  |  |  |  |  |  | [89] |
| P | Y | NA | | | | | | | | | [90] |
| P&N |  |  | Y | Y |  | Y |  |  |  |  | [91] |
| P |  | Y | Y |  |  | Y |  |  | Y |  | [92] |
| NA |  |  |  |  |  |  |  |  |  |  | [93] |
| P |  |  | Y |  |  | Y |  | Y |  |  | [94] |
| P |  |  | Y |  |  |  |  |  |  |  | [25] |
| P | Y |  | Y |  |  | Y |  |  |  |  | [95] |
| P | Y |  |  |  |  | Y |  |  |  |  | [96] |
| P |  |  |  |  |  | Y |  |  |  |  | [97] |
| P |  |  |  |  |  | Y |  |  |  |  | [98] |
| P |  | NA | | | | | | | | | [99] |
| NA |  |  | Y |  |  |  |  |  |  |  | [100] |
| P |  |  | Y |  |  |  |  |  | Y | Y | [101] |
| P |  |  |  |  |  | Y | Y | Y |  |  | [102] |
| NA |  |  |  |  |  |  |  |  |  |  | [103] |
| P |  |  | Y |  |  |  |  |  |  |  | [104] |
| P |  |  |  |  |  |  |  |  | Y | Y | [105] |
| NA |  | NA | | | | | | | | | [29] |
| P | Y | Y |  |  |  |  |  |  |  |  | [106] |
| NA |  |  | Y |  |  |  |  |  |  |  | [107] |
| P |  |  |  |  | Y | Y |  |  |  |  | [108] |
| N |  |  | Y | Y |  |  |  | Y |  |  | [109] |
| P | Y |  |  | Y |  |  |  | Y |  |  | [110] |
| P |  | NA | | | | | | | | | [111] |
| P |  |  | Y |  | Y | Y |  |  | Y |  | [112] |
| P |  |  | Y |  |  |  |  |  |  |  | [113] |
| P | Y |  | Y |  |  |  |  |  | Y |  | [114] |
| P&N |  |  |  | Y |  |  |  |  | Y |  | [115] |
| P |  | NA | | | | | | | | | [116] |
| P |  |  | Y |  |  |  | Y |  |  | Y | [144] |
| P |  | NA | | | | | | | | | [117] |
| P |  |  |  |  | Y | Y |  |  | Y |  | [118] |
| P |  |  |  |  |  |  |  | Y |  |  | [119] |
| P |  | NA | | | | | | | | | [120] |
| P |  | NA | | | | | | | | | [121] |
| P |  | Y | Y |  |  |  |  |  | Y |  | [122] |
| P |  |  |  |  |  |  |  |  |  |  | [123] |
| P |  | Y | Y |  |  |  |  |  |  |  | [124] |
| P |  |  | Y |  |  |  |  | Y |  |  | [125] |
| P |  | Y | Y | Y |  |  |  |  |  |  | [126] |
| P |  |  |  |  | Y | Y |  |  | Y |  | [127] |
| P |  | Y | Y |  |  |  |  | Y |  |  | [128] |
| P | Y |  | Y |  |  | Y |  |  |  |  | [129] |
| P&N |  | NA | | | | | | | | | [130] |
| NA |  |  | Y |  |  |  |  | Y |  |  | [131] |
| NA |  |  | Y |  |  |  |  |  |  |  | [132] |
| P |  | Y | Y |  |  | Y |  |  |  |  | [133] |
| P |  |  |  |  |  | Y |  |  |  |  | [134] |
| NA |  | NA | | | | | | | | | [135] |
| P |  | Y | Y |  |  | Y |  |  |  |  | [136] |
| P | Y |  | Y |  |  |  |  |  |  |  | [137] |
| P | Y |  | Y |  |  |  |  |  |  |  | [138] |
| P |  | Y | Y |  |  |  |  | Y |  |  | [139] |
| P | Y |  |  |  |  | Y | Y |  |  |  | [140] |
| NA |  |  |  | Y |  |  | Y |  |  |  | [141] |
| P |  | NA | | | | | | | | | [142] |
| P | Y |  | Y |  |  |  | Y |  | Y |  | [143] |
| NA |  |  | Y |  |  |  |  |  |  | Y | [145] |
| P |  | NA | | | | | | | | | [146] |
| P |  |  |  | Y |  | Y |  |  |  |  | [147] |
| NA |  |  |  |  |  | Y |  |  | Y |  | [148] |
| P | Y |  |  |  | Y | Y | Y | Y | Y |  | [149] |
| P |  | Y |  |  |  |  |  |  | Y |  | [150] |
| P |  | NA | | | | | | | | | [151] |
| P | Y | NA | | | | | | | | | [152] |
| P | Y | Y |  | Y |  |  |  | Y |  |  | [153] |
| P | Y |  | Y |  |  | Y |  |  |  |  | [154] |
| P | Y |  | Y |  |  | Y |  |  | Y |  | [155] |
| P | Y | NA | | | | | | | | | [156] |
| NA |  |  |  |  | Y | Y |  |  |  |  | [157] |
| NA |  |  | Y |  |  |  |  |  |  |  | [158] |
| P |  |  |  |  |  | Y |  |  | Y |  | [159] |
| NA |  | NA | | | | | | | | | [160] |
| NA |  | NA | | | | | | | | | [161] |
| P |  | NA | | | | | | | | | [162] |
| P |  |  | Y |  |  | Y |  |  |  | Y | [163] |
| NA |  |  |  |  |  |  |  |  | Y |  | [30] |
| P |  |  | Y | Y |  |  |  |  | Y |  | [164] |
| P |  |  |  |  |  |  |  |  | Y |  | [165] |
| P |  | NA | | | | | | | | | [166] |
| P | Y |  | Y |  | Y | Y |  |  |  |  | [167] |
| P | Y |  | Y |  |  |  |  |  |  |  | [168] |
| P | Y |  |  |  |  | Y |  |  |  |  | [32] |
| P |  | Y | Y |  |  |  |  |  | Y |  | [169] |
| P | Y |  |  |  |  | Y |  |  |  |  | [170] |
| P | Y | NA | | | | | | | | | [171] |
| NA |  | NA | | | | | | | | | [172] |
| NA |  | NA | | | | | | | | | [173] |
| P |  |  |  |  |  | Y |  |  |  |  | [174] |
| NA |  |  |  |  |  | Y |  |  |  |  | [175] |
| P | Y |  | Y |  |  |  |  |  |  |  | [176] |
| P |  |  | Y | Y |  |  |  |  | Y |  | [177] |
| P |  |  |  |  |  | Y |  |  |  |  | [178] |
| P |  | NA | | | | | | | | | [179] |
| P |  |  |  |  |  |  |  |  |  | Y | [180] |
| P |  |  |  |  |  | Y |  |  |  |  | [181] |
| P | Y |  | Y | Y |  | Y |  |  |  |  | [182] |
| P |  |  | Y |  |  | Y |  |  | Y |  | [183] |
| P |  | Y | Y |  |  | Y |  |  | Y |  | [184] |
| P | Y |  | Y |  |  |  |  | Y | Y |  | [185] |
| P |  |  | Y |  |  | Y |  |  |  |  | [186] |
| P | Y |  | Y |  |  | Y |  |  |  |  | [187] |
| P |  | NA | | | | | | | | | [188] |
